# Psychometric Properties of the Sexual Compulsivity Scale in Men Who Have Sex with Men in Spanish Population

**DOI:** 10.1007/s10461-022-03858-4

**Published:** 2022-09-20

**Authors:** Eduardo Ibáñez-Tomás, Rafael Ballester-Arnal, Marcel Elipe-Miravet, Àngel Gasch-Gallén

**Affiliations:** 1 Department of Physiatry and Nursing, University of Zaragoza, Zaragoza, Spain; 2 Servicio Aragonés de Salud (SALUD), Hospital Universitario Miguel Servet, Zaragoza, Spain; 3 Department of Basic and Clinical Psychology and Psychobiology, Universitat Jaume I de Castellón, Castellón de la Plana, Spain

**Keywords:** Sexual compulsivity, Assessment, Men who have sex with men, Psychometric properties

## Abstract

The Sexual Compulsivity Scale (SCS) has been translated, adapted and validated in general Spanish population, making its application difficult in certain groups, such as men who have sex with men (MSM). This paper evaluates the psychometric properties of the SCS in a sample of MSM in Spain. The SCS was administered to 881 participants. The factorial structure of the SCS was examined with an exploratory factor analysis (EFA) and confirmatory factor analysis (CFA). Both EFA and CFA confirmed a two-factor structure: (1) Interference of sexual behavior, and (2) Failure to control sexual impulses. Internal consistency was really good for the scale and also for both factors. The SCS also presented adequate psychometric properties. Thus, it is an appropriate measure for assessing sexual compulsivity in MSM, and a tool to be taken into account in future researches to reduce sexual risk behaviors in the MSM Spanish population.

## Resumen

La Escala de Compulsividad Sexual (SCS) ha sido traducida, adaptada y validada en población general española, siendo difícil su aplicación en determinados colectivos, como los Hombres que tienen Sexo con Hombres (HSH). Este trabajo evalúa las propiedades psicométricas del SCS en una muestra de 881 HSH de España. La estructura factorial del SCS se examinó con un Análisis Factorial Exploratorio (AFE) y un Análisis Factorial Confirmatorio (AFC). Tanto el AFE como el AFC confirmaron una estructura de dos factores: (1) Interferencia del Comportamiento Sexual y (2) Problemas en el Control de los Impulsos Sexuales. La consistencia interna fue muy buena, tanto para la escala como para los dos factores. El SCS también presentó adecuadas propiedades psicométricas. Por tanto, es una herramienta adecuada para evaluar la compulsividad sexual en HSH, y una escala a tener en cuenta en futuras investigaciones para reducir las conductas sexuales de riesgo de HSH en población española.

## Introduction

Sexual compulsivity (SC) is defined by sexual preoccupation and lack of sexual impulse control [1, 2]. It’s characterized by increased frequency of inappropriate or excessive sexual fantasies, behaviors, urges, and desires manifested, among others, by an excessive use of internet for sexual purposes, excessive pornography use, excessive masturbation or multiple casual sex anonymous partners [3]. The increase—in intensity and frequency—of these situations over time, has shown negative impacts in daily life and important health problems, such as low self-esteem (depression, anxiety, guilt, shame) [4–7], occupational difficulties (unemployment, financial problems), impaired social skills (loneliness, social isolation, divorce) [8, 9], and low use of condoms or other preventive methods, in order to prevent HIV and other STIs [9–11]. Some studies have mainly focused on the association between sexual compulsivity and sexual addictions [12–14] and the risk of HIV transmission and other sexually transmitted infections in different populations [5, 6, 10, 15, 16].

Different studies have found that specially men who have sex with men (MSM) are more likely than other populations to have difficulties with sexual compulsivity [17]. Previous research on this population, found that men scoring high on sexual compulsivity engage more frequently in unprotected sex and other risky sexual practices [4, 5, 7, 10], multiple causal sex partners [5, 16], more frequent substances abuse, such as alcohol, cocaine, methamphetamine or cannabis, among others [1, 4, 7, 18, 19], using sexual toys [10], having sexually transmitted infections [1] and also SC was found related to needs for increase self-control in chemsex [20].

Research has shed new light on the implications of Sexual Compulsivity in MSM, as for example the different implications that the role of affectivity plays in sexual behavior, depending on the sexual compulsivity scores on some MSM [21]. These findings shape that, without losing sight of the study of the SC as a predictive factor of unprotected intercourse [22] and their relation to substance use [18, 23], current trends on the study of the impact of sexual compulsivity on MSM health and behaviours have focused on SC relation to mental health and mental disorders, highlighting the importance of more deeply investigate on MSM [23, 24].

Some authors have proposed different questionnaires to evaluate sexual compulsivity. The Sexual Compulsivity Scale (SCS) was the first attempt to assess sexual compulsivity specifically [9], and today is the most widely used measure in research [25]. It’s a 10-item scale, rates each item ranging from 1 to 4 and yields total scores ranging from 10 to 40, higher scores indicating higher levels of sexual compulsivity. Some studies incorporating this instrument, have used a cutoff score of 24 or higher to indicate problems with sexual addiction [4, 16, 26].

The SCS has been translated and validated in different languages and populations such as Brazilian [27], or Spanish [28], among others. On the basis of this first instrument, other questionnaires have been developed to assess compulsive sexual behavior. One of them is the Compulsive Sexual Behavior Inventory (CSBI-22) [29, 30], that assesses two factors: control and violence. Recently, Bőthe et al. [31] have also designed a scale to assess Compulsive Sexual Behavior Disorder (CSBD-19). Its 19 items assess control, salience, relapse or unsuccessful efforts to reduce or cease sexual activity, absence or decrease in sexual satisfaction, and negative consequences. Other less known scales are the Individual-Based Compulsive Sexual Behavior Scale (I-CSB), focused on four core symptoms in the diagnosis of compulsive sexual behavior [32]: control, unwanted consequences, negative affect, and affect regulation; the Compulsive Sexual Behavior Consequences Scale (CSBCS) designed to assess the consequences of compulsive sexual behavior (e.g., intimate relationships, risky sexual behaviors, interpersonal relationships, etc.) [33]; or the Cognitive and Behavioral Outcomes of Sexual Behavior Scale (CBOSBS) [34]. And many other questionnaires have been developed to assess constructs similar to sexual compulsivity such as sex addiction or hypersexuality. In addition, also based on the SCS, some scales have been developed that assess more specific constructs such as the use of online pornography. An example is the Cyberporn Compulsivity Scale (CCS) [35].

In this paper, we have focused working with the SCS. The main reasons are: (1) it was the first questionnaire to assess the SC, (2) nowadays it is the most used for the researchers, (3) most of the posterior questionnaires have used the SCS as a base, in order to create or evaluate new areas, (4) it is the shortest, and it has a good statistical evidence [25], (5) it has been translated to several languages, included Spanish, and (6) it has also been applicated in different sample populations, such as heterosexual men and women [36], college students [10, 37, 38], male escorts [16, 39], HIV-positive men and women [4, 5], as well as a men who have sex with other men (MSM) [4, 40, 41].

However, for the Spanish population, the SCS has been translated, adapted and validated only in the general Spanish population [28], making its application difficult in certain populations such as MSM. To the best of our knowledge, there is no previous studies on the validation of this instrument in this specific population in Spain. Thus, our purpose was to evaluate the psychometric properties of the SCS in a sample of MSM in Spain.

## Methods

### Design

A cross-sectional descriptive quantitative study, based on an online survey of sexual compulsivity in MSM in Spain. A convenience sampling method was used.

### Participants

Participants were individuals who self-identified as men. In order to be included in the sample, participants had to meet three requirements: (1) they must be between 16 and 75 years old, (2) residents in Spain, and (3) declare that they had sexual relations with other men in the previous 12 months. Participation in the study was voluntary. Each participant approved an online informed consent document prior to taking participation in the study.

The final study sample consisted of 881 MSM. The ages of the participants ranged between 16 and 74 years (M = 33.13; SD = 9.99). Most of the participants were born in Spain (72.7%; n = 641), a 22.9% were born in America (n = 202), and the other 4.2% were from Europe (n = 32), Asia (n = 3) and Africa (n = 3). About their sexual orientation, an 85.7% considered themselves as homosexuals, a 13.3% as bisexuals and only a 1% as heterosexuals. Furthermore, 98.9% (n = 871) of the participants were cissexual men, whereas only a 1.1% (n = 10) were transgender men. Most of them lived with their family or with friends (42.8%), a 35% were living alone, and finally a 22.2% were living with their partners/husbands. Related to the use of ICT’s (Information and Communications Technologies), a 29.2% of our sample affirmed that they always used an App or web pages for finding a sexual partner, a 37.5% admitted they used those methods almost all times, a 27.2% only sometimes, and finally a 6.1% of our sample have never used technologies to find a sexual partner.

### Instruments

First, an ad hoc questionnaire was administered to assess some socio-demographic variables such as sex assigned at birth, age, place of birth, academic level, employment status (employed, unemployed, student, retired), income (more than 1200 euros/month, between 700 and 1200/month and less than 700 euros per month), living situation (alone, with a partner or with other people), size of city of residence, and sexual orientation and gender identity (heterosexual, bisexual, gay, cisexual man, trans man, other).

Then, the validated Spanish version [28] of the SCS [9] was administered. This scale consists of 10 Likert-type items, measured on a scale ranging from 1 (not at all) to 4 (always). Total score ranges between 10 and 40. Cronbach´s alpha coefficient for total scale was α = 0.837. The factorial analysis of the Spanish version for general population resulted in two factors [28]: (1) Interference of sexual behavior (items 1, 2, 3, 4 and 10; α = 0.72), that includes questions about the interference that sex has in several life aspects of participants, and (2) Failure to control sexual impulses (items 5, 6, 7, 8, and 9; α = 0.79), which includes items that refer to concerns and difficulties that people have or experience about issues related to sex and its impulses. An English and Spanish version of SCS are presented in Appendix 1 and Appendix 2.

### Procedure

A web-based was made using Google Forms. Data were collected through an online survey from December 2019 to January 2020. It was disseminated with the support of non-governmental organizations dedicated to HIV prevention in Spain. We also collaborated with Grindr, a mobile geo-social application, aimed at gay audience, which enables its users to locate and communicate with other gays, bisexuals, and transgender individuals in the vicinity. On their mobile app, they published the information of the study and included a shortened link for easier access to the online survey. In the survey was said that participants would only answer if they were of legal age, that for sexual matters in Spain is 16 years old or older. Anonymity and confidentiality of participants were guaranteed during the process.

The research was carried out with the approval of the Research Ethics Committee of the Government of Aragon, Spain (CEICA, C.P.-C.I. PI18/327). The research also fulfilled the rules and ethical principles of the Declaration of Helsinki.

### Statistical Analyses

Different statistical software programs were used in this research. The SPSS (version 25) was used to carry out descriptive analyses of the sociodemographic data and for checking the items’ correlations with the two factors.

To obtain the factor structure of the scale, an Exploratory Factor Analysis (EFA) was performed with the data of 401 participants. For that purpose, the statistical software Mplus (version 7.4) was selected [42]. This software makes it possible to create structural models with categorical variables and to obtain a factorial structure based on polychoric correlations using the robust WLSMV estimator [42, 43]. In the EFA, the ideal number of factors was extracted from the eigenvalues, the Factor Determinacy Index (FDI) and a set of goodness-of-fit (GOF) statistics.

To corroborate the EFA structure, a Confirmatory Factor Analysis (CFA) was run using the RStudio software, concretely the Lavaan package, with the data of the other 480 participants. Furthermore, analyses of invariance (configural, metric, scalar and strict) were performed in order to confirm the structure across income level groups using the same package.

The analysis of the GOF was performed with the following indices: Satorra-Bentler chi-square (Chi^2^), statistical probability (p), Root Mean Square Error of Approximation (RMSEA), Comparative Fit Index (CFI), Tucker-Lewis Index (TLI), Incremental Fit Index (IFI), Standardized Root Mean Square Residual (SRMR) and finally the Modification Index (MI) and the Expected Parameter Change (EPC) [44]. An acceptable overall fit corresponds to values of RMSEA < 0.06, SRMR < 1, and CFI, TLI and IFI > 0.90 [45–47]. Excellent values correspond to values of CFI, TLI and IFI > 0.95, RMSEA < 0.05, and SRMR < 0.08 [45, 46].

Finally, the RStudio software was also used to calculate the reliability of the scale and their dimensions. According to Viladrich et al. [48], for ordinal items should be used "ordinal omega" if they are non-tau equivalent, and "ordinal alpha" if they are tau equivalent. Thus, for the global test reliability and for each dimension reliability was used the “coefficient alpha” package [49], given that this package provides both reliability indices.

## Results

### Exploratory Factor Analysis (EFA)

To determine the empirical structure of the instrument, an exploratory factor analysis (EFA) was performed using the statistical software MPlus 7.4 [42]. For this type of analysis, the oblique method with Geomin rotation was used, because it provides small cross-loadings, minimizing variables complexity and producing a cleaner factor structure [50]. We also used the Weighted Least Squares Mean and Variance Adjusted (WLSMV) estimator because it is the most suitable for categorical variables [51].

**Fig. 1:**
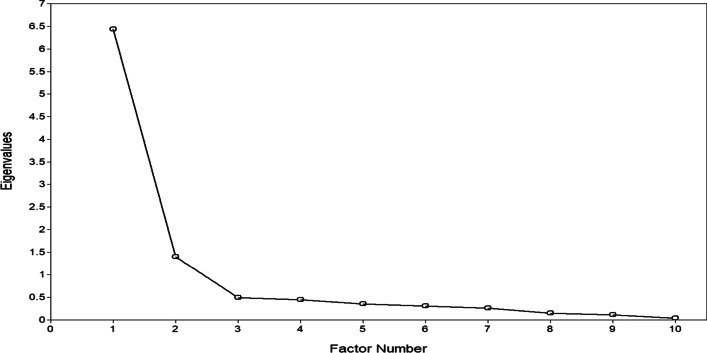
Exploratory factorial analysis scree plot

The Eigenvalue scree plot shows that, for the EFA, the 2-factor structure best fits the data (Fig. 1).

Furthermore, fit statistics results reflect the same conclusion, given that the unidimensional model has a RMSEA > 0.60 and a SRMR > 1.0 (Table 1); and models with 3 or more components have factors in which no items have a factor loading above 0.30, minimum acceptable value to belong to a factor [52].

**Table 1:** EFA fit measures

Factors	χ^2^	df	*p*	CFI	TLI	RMSEA	SRMR
1	242.736	35	< 0.001	0.931	0.911	0.122	0.108
**2**	**36.563**	**26**	**0.082**	**0.996**	**0.994**	**0.032**	**0.028**
3	23.733	18	0.164	0.998	0.996	0.028	0.020
4	9.683	11	0.559	1.00	1.00	0.00	0.011

Bold characters represents the selected model

Table 2 shows that the first factor is made up of 5 items (1, 2, 3, 4 and 10) and has an eigenvalue of 6.43. The second factor is also made up of 5 items (5, 6, 7, 8 and 9) and has an eigenvalue of 1.40. The two Factor Determinacy Indices have really high values (FDI > 0.80), both above the minimum acceptability value for the quality of the factor score [53].

**Table 2:** EFA Factorial Loadings for the 2-factor model

Items	F1	F2
1.My sexual appetite has gotten in the way of my relationships	0.795	
2.My sexual thoughts and behaviours are causing problems in my life	0.897	
3.My desires to have sex have affected my daily life	0.923	
4.I sometimes fail to meet my commitments and responsibilities because of my sexual behaviours	0.758	
5.I sometimes get so horny that I could lose control		0.780
6.I find myself thinking about sex while at work		0.818
7.I feel that my sexual thoughts and feelings are stronger than I am		0.953
8.I have to struggle to control my sexual thoughts and behaviours		0.917
9.I think about sex more than I would like to		0.813
10.It has been difficult for me to find sex partners who desire having sex as much as I want to	0.757	
Factor Determinacy Index	0.968	0.977

### Confirmatory Factor Analysis (CFA)

To ratify the factor structure of the SCS, a confirmatory factor analysis was performed using Lavaan package [54]. Again, the WLSMV estimator was used. Based on the results obtained in the EFA, four models were compared (see Table 3).

- The first model (M1) analyzed a unifactorial structure, as a reference model, with all the items that the EFA included in the scale structure (unifactorial model).
- The second model (M2) exactly replicated the factor structure derived from the EFA (two correlated first-order factors).
- Since fit statistics in M2 were not good enough, some adjustments were made. In this third model (M3) item 10 was moved from Factor 1 to Factor 2, following the Modification Index (MI) and the Expected Parameter Change (EPC).
- As happened before, M3 had an inappropriate RMSEA value. Following the MI and the EPC indications, a fourth model (M4) was run, correlating the residual covariances of items 10 and 9. This was the final model, given that all fit statistics were acceptable or very good and the MI and the EPC did not suggest more improvements for this model.

**Table 3:** Goodness of fit indexes for the CFA

	χ^2^	df	CFI	TLI	IFI	RMSEA (90% CI)	SRMR
Model 1	239.341***	35	0.927	0.907	0.911	0.110 (0.097–0.124)	0.075
Model 2	170.666***	34	0.951	0.936	0.931	0.091 (0.078–0.105)	0.067
Model 3	103.604***	34	0.975	0.967	0.963	0.065 (0.051–0.080)	0.051
**Model 4**	88.184***	**33**	**0.980**	**0.973**	**0.975**	**0.059 (0.044–0.074)**	**0.047**

* p < 0.05, ** p < 0.01, *** p < 0.0001

Considering the content of the items, the first factor, which includes items 1, 2, 3 and 4, is called "Interference in sexual behavior" and the second factor, which includes items 5, 6, 7, 8, 9 and 10, is called "Failure to control sexual impulses". The variance explained for each factor is 43.9% and 56.2%, respectively.

**Fig. 2:**
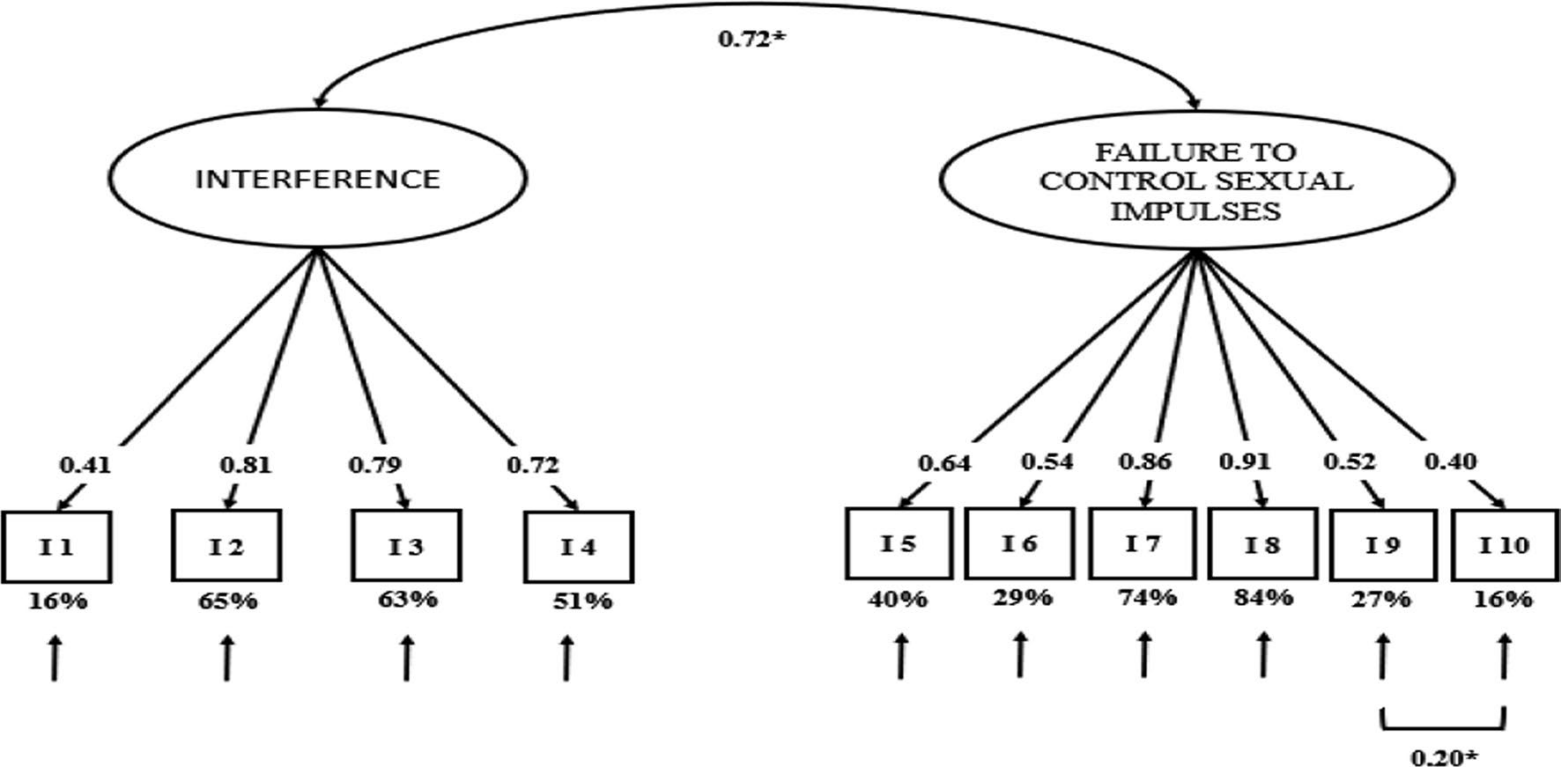
Confirmatory factor analysis for the SCS. Coefficients are reported as standardised. Endogenous variables were significant at *p* < 0.001. Variable r^2^ is expressed as a percentage outside the variable boxes. * p < 0.05

In M4, the index IFI = 0.975; the TLI = 0.973; and the CFI = 0.980. All three indexes achieve very high values, above the strictest criteria for the excellence. The same occurs with the SRMR = 0.047, achieving also an excellent value. Finally, the RMSEA = 0.059, which has an acceptable value, bellow the limit for acceptability. The model can be seen in Fig. 2.

Regarding the assessment of invariance, the results show that the SCS is invariant for the income level, either for the structure, factor loadings as well as for intercepts, thresholds and residuals (see Table 4). Regarding the RMSEA values for the evaluation of the four invariances, the metric, scalar and strict models have values that are below the established cut-off point (RMSEA < 0.05) for excellent values [55], whereas the configural model has an acceptable value (RMSEA = 0.054). The IFI, CFI and TLI values in the four models analyzed are above the accepted cut-off point, being also excellent values [47]. Finally, SRMR is excellent in all four invariance models. As the evaluation of invariances are supported at all levels, the next step was to compare the nested models. As all comparisons are significant, we can assure that our scale does not change at all depending on its structure, the factor loading, the thresholds and the residuals for both groups.

**Table 4:** Factorial invariance of CFA across groups

	χ^2^	df	IFI	CFI	TLI	RMSEA	SRMR	Comparison	Δχ2	Δ*df*	*p*
C	79.02***	66	0.990	0.990	0.986	0.054	0.041	C-M	11.16	18	0.888
M	84.81***	84	0.991	0.991	0.990	0.045	0.045	M-S	3.63	8	0.889
S	88.10***	92	0.993	0.993	0.993	0.039	0.042	C-S	15.56	26	0.946
St	95.56***	102	0.994	0.994	0.994	0.039	0.044	C-St	24.15	36	0.934

*C* configural invariance, *M* metric invariance, *S* scalar invariance, *St* strict invariance, Δχ2 was calculated according to Satorra-Bentler Chi-Square Difference Testing for WLSMV estimator. * p < 0.05, ** p < 0.01, *** p < 0.001

### Descriptive Data and Reliability

Table 5 shows the means, standard deviations, asymmetry, and kurtosis for each of the items and for the two factors of the scale, in addition to the reliability of each dimension.

**Table 5:** Descriptive statistics and reliability indexes for items and factors of the SCS

	Range	*M (SD)*	Skewness	Kurtosis	Reliability indexes		
					α (CI)	Ω (CI)	I–F *r*
*F1—Interference of sexual behavior*	4–16	5.90 (2.425)	1.708	2.901	0.86 (0.85–0.88)	0.87 (0.85–0.88)	NA
Item 1	1–4	1.60 (0.831)	1.285	0.856	NA	NA	0.730
Item 2	1–4	1.42 (0.772)	1.930	3.079	NA	NA	0.731
Item 3	1–4	1.53 (0.789)	1.509	1.732	NA	NA	0.786
Item 4	1–4	1.34 (0.711)	2.289	4.853	NA	NA	0.641
*F2—Failure to control sexual impulses*	6–24	10.07 (4.05)	1.222	1.057	0.90 (0.89–0.91)	0.90 (0.89–0.91)	NA
Item 5	1–4	1.55 (0.834)	1.46	1.287	NA	NA	0.668
Item 6	1–4	1.93 (0.951)	0.745	− 0.426	NA	NA	0.718
Item 7	1–4	1.57 (0.855)	1.397	1.022	NA	NA	0.773
Item 8	1–4	1.47 (0.793)	1.66	1.941	NA	NA	0.723
Item 9	1–4	1.91 (1.006)	0.753	− 0.636	NA	NA	0.754
Item 10	1–4	1.63 (.917)	1.338	0.710	NA	NA	0.643

*α* ordinal alpha, *Ω* ordinal omega, *NA* not applicable, I–F *r* = corrected item–factor correlation

For factor 1, values range between 4 and 16, being 4 an absence of interference and 16 a high interference. For items 2, 3 and 4, our sample seems to have a very low interference, whereas item 1 generates a little bit more interference than the other three items.

In the second factor, values range between 6 and 24 (the higher the score is, the more problems they have to control their sexual impulses). Our sample seems to have more complications to control their impulses in thinking about sex in their work and in general, whereas they do not have to fight too much to control their sexual thoughts and behaviours.

Regarding the internal consistency, and concretely ordinal alpha reliability index, factors 1 and 2 achieve values of α = 0.86 and α = 0.90, respectively (see Table 5). Moreover, the reliability for factors 1 and 2 evaluated with the omega ordinal coefficient achieve values of Ω = 0.87 and Ω = 0.90, respectively. About the global test reliability, α = 0.88 and Ω = 0.88. Both alpha and omega ordinal indices have practically the same values for both factors and for the global reliability. Additionally, the item-factor correlation was calculated, reaching in all cases high and significant values (I–F *r* > 0.60; *p* < 0.001).

## Discussion

This study aimed to determine the construct validity and the psychometric properties of the SCS in Spanish MSM. Two factors emerged from the analysis, according to previous use of this instrument in Spain [28]. Considering the content of the items, the first factor, which includes items (1) “My sexual appetite has gotten in the way of my relationships”, (2) “My sexual thoughts and behaviours are causing problems in my life”, (3) “My desires to have sex have affected my daily life” and (4) “I sometimes fail to meet my commitments and responsibilities because of my sexual behaviours”, is called "Interference of sexual behaviours" and explains the 43.9% of the model variance. And the second factor, which includes items (5) “I sometimes get so horny that I could lose control”, (6) “I find myself thinking about sex while at work”, (7) “I feel that my sexual thoughts and feelings are stronger than I am”, (8) “I have to struggle to control my sexual thoughts and behaviours”, (9) “I think about sex more than I would like to” and (10) “It has been difficult for me to find sex partners who desire having sex as much as I want to”, is called "Failure to control sexual impulses" and explains the 56.2% of the model variance.

The original SCS was conducted with a sample of sexually active men who considered themselves homosexuals (n = 160) [9]. Later, Kalichman and Rompa [6] used the scale in two different samples: (a) one of gay men (n = 296) and (b) another of African American men (n = 60) and African American women (n = 98) from inner-city areas and low-income groups. Nevertheless, factor analysis was not performed in the original studies [6, 9]. We know only that the original scale showed high levels of internal consistency (reliability): α = 0.89 in the first study and α = 0.86 for gay men and α = 0.87 for African American men and women in the second study.

In further studies, it found a two-factor solution for the SCS [1, 38]. Both called these factors in the same way: Factor 1 was called “social disruptiveness” and Factor 2 was called “personal discomfort”. However, some items (2, 5, 6, and 7) were grouped on opposite factors on these studies. In Kalichman and Cain’s study [1], which used a sexually transmitted infection treatment-seeking sample population, as result of the principal component analysis using a VARIMAX rotation, the first factor included items 1 to 4 that represented a social disruptiveness dimension and accounted for 50.8% of the variance. The second factor included items 5 to 10 that represented a personal discomfort dimension to sexual compulsivity and accounted for 10.7% of variance. Comparisons of men and women on the two sexual compulsivity dimensions showed that men did not differ from women on the social disruptiveness factor. However, men scored significantly higher than women on the personal discomfort factor.

In other subsequent studies such as the validation of the questionnaire in China with a sample of sexually active men, two factors were obtained [56]. A first factor explaining 34.5% of the total variance including items 5, 6, 7, 8, and 9; and a second factor explaining items 1, 2, 3, and 10. Item 4 was removed from the questionnaire as it saturated almost equally in both factors. The authors named the factors found "Controllability" and "Functional Consequences", labels very similar to those proposed by us. However, if analyzed carefully, the factor loadings of item 10 are also very similar: 0.38 in factor 1 and 0.41 in factor 2, in which they propose to include it.

In the validation conducted by Scanavino et al. [27], with a sample of 153 Brazilian men with excessive sexual drive according to ICD-10 criteria and who met the criteria for sex addiction, they offer a single 10 items factor solution that explains 69.2% of variance and has a reliability of α = 0.95.

Regarding the only validation in Spain prior to the study we present, carried out with men and women from the general population [28], the authors also found two factors, but they were named differently from the one proposed by Kalichman and Cain [1]. Factor 1 was labeled as “Interference of sexual behavior”, while Factor 2 was called “Failure to control sexual impulses”. Both factors reflected variations in factor loadings of individual items compared to those obtained in the study by McBride et al. [38], which was conducted with a sample of young people. However, these results were similar to those obtained by Kalichman and Cain [1]. There was only a difference in factor loading of item 10, “It has been difficult for me to find sex partners who desire having sex as much as I want to”. This item belongs to the Personal discomfort factor in the study of Kalichman and Cain [1], while it belongs to the Interference of sexual behavior factor in the study of Ballester-Arnal et al. [28].

In the present study with a sample of spanish MSM, we have also obtained two factors that have been labelled identically than in the previous study with Spanish general population: “Interference of sexual behaviours” and “Failure to control sexual impulses”. In general, the composition of the factors is also identical except for item 10 "It has been difficult for me to find sex partners who desire having sex as much as I want to", which in the present work becomes part of factor 2 "Failure to control sexual impulses". Interestingly, it is the only item that also differentiated the validation of Kalichman and Cain [1] from that of Ballester-Arnal et al. [28].

The differences in terms of the place occupied by item 10 in different studies may have several explanations. One of them has to do with the type of statistical analysis performed. Of all of the validations we have discussed, only two provide EFA data [1, 56]. In our study, also the Exploratory Factor Analysis (EFA) offers two factors in which item 10 appears grouped with 1, 2, 3 and 4 in the "Interference of sexual behaviors" factor. However, the best model offered by the Confirmatory Factor Analysis best places item 10 in factor 2 of "Failure to control sexual impulses". A second explanation has to do with cultural differences. The construct of sexual compulsivity seems to be sensitive to these differences and it is possible that the structure of the construct and the weight of the different dimensions is also different in different cultures. Thus, in the study of Liao et al. [56], the Controllability Subscale but not the Functional Consequences Subscale was associated with self-reported STI. According to these authors, Chinese culture emphasizes on self-control and harmony and pays less attention to individualism and personal comfort. Because of this, it’s reasonable that controllability and functional consequences replaces self-disruption and personal comfort as new constructs of the SCS. The data indicate that comparisons between studies conducted in different cultures should be made with caution for this reason. The third explanation has to do with the composition of the samples evaluated. Some studies work with a general population composed of men and women, others with university students, others with patients attending sexually transmitted infection treatment clinics, with a homosexual population (gays and lesbians) or with men who have sex with men. The structure of the questionnaire and the variance explained by each factor may depend on the type of sample. The fact that in our study item 10 "It has been difficult for me to find sex partners who desire having sex as much as I want to" is grouped with other items that are more related to failure to control sexual urges than to interference with sexual behavior could be related to two issues. It is possible that perhaps sexual frequency or sexual responsiveness in MSM may be higher than in the general population where the majority is heterosexual. Therefore, it would be more complicated for a high frequency to interfere with the couple's life and it would become an impulse control item like the others. But another alternative is that in the previous Spanish validation [28] there was a large female sample and women tend to score lower in sexual frequency, so that in the present study, which analyzes only the behavior of men, this frequency would be higher, not because they are MSM but because they are men.

In our study with MSM, first factor, showed in general low scores, which suggests, contrary to other populations [25, 36], that in MSM specifically, SC produces low interference in the daily life. Specific factors, capable to break down this balance, should be investigated in order to better understand and also manage unbalance situations, playing an important role on the development and maintenance of sexually compulsive behavior in this population [17]. Second factor in our sample showed the existence of difficulties to control impulses in thinking about sex at work and in general. Some research suggest the need to explore the impact of negative mood on the tendency to specifically seek out sex as a form of distraction, for validation or to obtain emotional support [24]. Rooney et al. [19] found sexual compulsivity associated to depression and anxiety, These findings led us to focus on the important role of the impulse control of SC in MSM, when it comes to design specific interventions related to self-care.

Finally, in terms of reliability, the factors resulting from this study present a high internal consistency: α = 0.86 for the factor Interference of sexual behaviorus and α = 0.90 for the factor Failure to control sexual impulses. In the validation with Spanish general population [28], internal consistency in total scale was α = 0.84. Our result is very similar than the pilot study of the scale undertaken by Kalichman et al. [9], in which participants were sexually active men who considered themselves homosexual (α = 0.89). It has also been similar than the original study by Kalichman and Rompa [6] of gay men (α = 0.86) and of primarily African American men and women in inner-city areas on low incomes (α = 0.88). In the study of Dodge et al. [10] among students aged 18 to 25 years old, reliability was α = 0.82.

In summary, the SCS shows excellent psychometric data in its application with men who have sex with men in Spain and therefore shows its usefulness to be applied with this population.

### Limitations

This study validated the Sexual Compulsivity Scale among MSM population in Spain. Nevertheless, it has some limitations that ought to be addressed in future research. Firstly, the type of sampling for convenience does not allow the generalization of results to the MSM national Spanish population. Although the number of participants is the largest analyzed in this population, we need to understand our findings as a first exploratory approach in this field among MSM. Secondly, most of the participants were recruited by a dating application, therefore, may not be representative of general MSM Spanish population. Thirdly, in this research we have not checked for convergent or discriminant validity. Although the SCS has good evidence of convergent validity [25], it has not been checked in our sample, that is, in a Spanish MSM population. That could be an important point to be explored in the future. Furthermore, the use of a self-report instrument may have resulted in under-reporting of some sexual behaviors to match social desirability.

Thereby, future studies could be focused on the representativeness of the sample, improving and complementing different strategies for the participants’ recruitment, in order to faithfully represent the MSM population.

## Conclusion

Sexual Compulsivity has been related to different disorders and health problems. To sum up, our study is the first to validate SCS among an identified sample of MSM in Spain. This research can make an important scientific contribution to health promotion and STIs and HIV prevention among MSM population. The inclusion of this scale in future studies can highlight a need to implement more effective strategies to reduce sexual compulsivity and related sexual risk behaviours.

## Appendix 1 (English version)

The following is a series of statements about your sexual behavior, so that you can answer to what extent these statements are characteristic of you.

**Table:**

Items			
1. My sexual appetite has gotten in the way of my relationships			
□ Not at all	□ Sometimes	□ Quite often	□ Always
2. My sexual thoughts and behaviours are causing problems in my life			
□ Not at all	□ Sometimes	□ Quite often	□ Always
3. My desires to have sex have affected my daily life			
□ Not at all	□ Sometimes	□ Quite often	□ Always
4. I sometimes fail to meet my commitments and responsibilities because of my sexual behaviours			
□ Not at all	□ Sometimes	□ Quite often	□ Always
5. I sometimes get so horny that I could lose control			
□ Not at all	□ Sometimes	□ Quite often	□ Always
6. I find myself thinking about sex while at work			
□ Not at all	□ Sometimes	□ Quite often	□ Always
7. I feel that sexual thoughts and feelings are stronger than I am			
□ Not at all	□ Sometimes	□ Quite often	□ Always
8. I have to struggle to control my sexual thoughts and behaviours			
□ Not at all	□ Sometimes	□ Quite often	□ Always
9. I think about sex more than I would like to			
□ Not at all	□ Sometimes	□ Quite often	□ Always
10. It has been difficult for me to find sex partners who desire having sex as much as I want to			
□ Not at all	□ Sometimes	□ Quite often	□ Always

## Appendix 2 (Spanish version)

A continuación, te presentamos una seria de afirmaciones sobre tu comportamiento sexual para que respondas en qué medida estos enunciados son característicos en ti.

**Table:**

Items			
1. Mi gran apetito sexual ha sido un obstáculo en mis relaciones			
□ Nada	□ Algo	□ Bastante	□ Mucho
2. Mis pensamientos y comportamientos sexuales me están causando problemas en la vida			
□ Nada	□ Algo	□ Bastante	□ Mucho
3. Mis deseos de tener sexo han afectado en mi vida cotidiana			
□ Nada	□ Algo	□ Bastante	□ Mucho
4. A veces no consigo cumplir con compromisos y responsabilidades a causa de mis comportamientos sexuales			
□ Nada	□ Algo	□ Bastante	□ Mucho
5. A veces llego a ponerme tan caliente que podría perder el control			
□ Nada	□ Algo	□ Bastante	□ Mucho
6. Me sorprendo a mí mismo pensando sobre sexo en el trabajo			
□ Nada	□ Algo	□ Bastante	□ Mucho
7. Siento que mis pensamientos y sensaciones sexuales son más fuertes que yo			
□ Nada	□ Algo	□ Bastante	□ Mucho
8. Tengo que luchar para controlar mis pensamientos y comportamientos sexuales			
□ Nada	□ Algo	□ Bastante	□ Mucho
9. Pienso en sexo más de lo que me gustaría			
□ Nada	□ Algo	□ Bastante	□ Mucho
10. Me ha resultado difícil encontrar parejas sexuales que desearan tener sexo tanto como yo			
□ Nada	□ Algo	□ Bastante	□ Mucho

## References

[1] Kalichman S, Cain D (2004). The relationship between indicators of sexual compulsivity and high risk sexual practices among men and women receiving services from a sexually transmitted infection clinic. J Sex Res.

[2] Quadland M (1985). Compulsive sexual behavior: definition of a problem and an approach to treatment. J Sex Marital Therapy.

[3] Parsons J, Kelly B, Bimbi D, DiMaria L, Wainberg M, Morgenstern J (2007). Explanations for the origins of sexual compulsivity among gay and bisexual men. Arch Sex Behav.

[4] Benotsch E, Kalichman S, Kelly J (1999). Sexual compulsivity and substance use in hiv-seropositive men who have sex with men. Addict Behav.

[5] Benotsch G, Kalichman S, Ste E (2001). Sexual compulsivity in HIV-positive men and women: prevalence, predictors, and consequences of high-risk behaviors. Sex Addict Compuls.

[6] Kalichman S, Rompa D (2001). The sexual compulsivity scale: further development and use with HIV-positive persons. J Pers Assess.

[7] Semple S, Zians J, Grant I, Patterson T (2006). Sexual compulsivity in a sample of HIV-positive methamphetamine-using gay and bisexual men. AIDS Behav.

[8] Black DW, Kehrberg LL, Flumerfelt DL, Schlosser SS (1997). Characteristics of 36 subjects reporting compulsive sexual behavior. Am J Psychiatry.

[9] Kalichman S, Johnson J, Adair V, Rompa D, Multhauf K, Kelly J (1994). Sexual sensation seeking: scale development and predicting AIDS-risk behavior among homosexually active men. J Pers Assess.

[10] Dodge B, Reece M, Cole S, Sandfort T (2004). Sexual compulsivity among heterosexual college students. J Sex Res.

[11] McCoul M, Haslam N (2001). Predicting high risk sexual behaviour in heterosexual and homosexual men: the roles of impulsivity and sensation seeking. Personal Individ Differ.

[12] Ballester-Arnal R, Castro-Calvo J, Giménez-García C, Gil-Juliá B, Gil-Llario M (2020). Psychiatric comorbidity in compulsive sexual behavior disorder (CSBD). Addict Behav.

[13] Ballester-Arnal R, Castro-Calvo J, García-Barba M, Ruiz-Palomino E, Gil-Llario M (2021). Problematic and non-problematic engagement in online sexual activities across the lifespan. Comput Hum Behav.

[14] Gil-Llario M, Gil-Juliá B, Morell-Mengual V, Cárdenas-López G, Ballester-Arnal R (2021). Analysis of demographic, psychological and cultural aspects associated with the practice of sexting in Mexican and Spanish adolescents. Int J Intercult Relat.

[15] Chumakov E, Petrova N, Kraus S (2019). Compulsive sexual behavior in HIV-infected men in a community based sample, Russia. Sex Addict Compuls.

[16] Parsons T, Bimbi D, Perry NHJ (2001). Sexual compulsivity among gay/bisexual male escorts who advertise on the internet. Sex Addict Compuls.

[17] Chaney M, Burns-Wortham C, Birchard T, Benfield T (2018). Sexual compulsivity and men who have sex with men (MSM). The Routledge international handbook of sexual addiction.

[18] Garner A, Shorey R, Anderson S, Stuart G (2020). Risky sexual behaviors among men in residential treatment for substance misuse: the role of compulsive sexual behavior. Sex Addict Compuls.

[19] Rooney B, Tulloch T, Blashill A (2017). Psychosocial syndemic correlates of sexual compulsivity among men who have sex with men: a meta-analysis. Arch Sex Behav.

[20] Evers Y, Hoebe C, Dukers-Muijrers N, Kampman C, Kuizenga-Wessel S, Shilue D (2020). Sexual, addiction and mental health care needs among men who have sex with men practicing chemsex—a cross-sectional study in the Netherlands. Prevent Med Rep.

[21] Grov C, Golub S, Mustanski B, Parsons J (2010). Sexual compulsivity, state affect, and sexual risk behavior in a daily diary study of gay and bisexual men. Psychol Addict Behav.

[22] Ni Y, Liu H, Gong R, Shi M, Zhang S, Wang S (2021). The role of sexual compulsivity in unprotected intercourse among STI patients in Shanghai, China. BMC Public Health.

[23] Achterbergh R, van Rooijen M, van den Brink W, Boyd A, de Vries H (2020). Enhancing help-seeking behaviour among men who have sex with men at risk for sexually transmitted infections: the syn.bas.in randomised controlled trial. Sex Transm Infect..

[24] Janssen E, Prause N, Swinburne Romine R, Raymond N, MacDonald A, Coleman E (2020). Sexual responsivity and the effects of negative mood on sexual arousal in hypersexual men who have sex with men (MSM). J Sex Med.

[25] Hook J, Hook J, Davis D, Worthington E, Penberthy J (2010). Measuring sexual addiction and compulsivity: a critical review of instruments. J Sex Marital Therapy.

[26] Cooper A, Delmonico D, Burg R (2000). Cybersex users, abusers, and compulsives: new findings and implications. Sex Addict Compuls.

[27] Scanavino M, Ventuneac A, Rendina H, Abdo C, Tavares H, Amaral M (2014). Sexual compulsivity scale, compulsive sexual behavior inventory, and hypersexual disorder screening inventory: translation, adaptation, and validation for use in Brazil. Arch Sex Behav.

[28] Ballester-Arnal R, Gómez-Martínez S, Gil-Llario M, Salmerón-Sánchez P (2013). Sexual compulsivity scale: adaptation and validation in the Spanish population. J Sex Marital Therapy.

[29] Coleman E, Miner M, Ohlerking F, Raymond N (2001). Compulsive sexual behavior inventory: a preliminary study of reliability and validity. J Sex Marital Therapy.

[30] Miner M, Coleman E, Center B, Ross M, Rosser B (2006). The compulsive sexual behavior inventory: psychometric properties. Arch Sex Behav.

[31] Bőthe B, Potenza M, Griffiths M, Kraus S, Klein V, Fuss J (2020). The development of the Compulsive Sexual Behavior Disorder Scale (CSBD-19): an ICD-11 based screening measure across three languages. J Behav Addict.

[32] Efrati Y, Mikulincer M (2018). Individual-based compulsive sexual behavior scale: its development and importance in examining compulsive sexual behavior. J Sex Marital Therapy.

[33] Muench F, Morgenstern J, Hollander E, Irwin T, O'Leary A, Parsons J (2007). The consequences of compulsive sexual behavior: the preliminary reliability and validity of the compulsive sexual behavior consequences scale. Sex Addict Compuls.

[34] McBrid K, Reece M, Sanders S, Terri D, Clive M, William L, Sandra L (2010). Cognitive and behavioral outcomes of sexual behavior scale. Handbook of sexuality-related measures.

[35] Abell J, Steenbergh T, Boivin M (2006). Cyberporn use in the context of religiosity. J Psychol Theol.

[36] Muise A, Milhausen RR, Cole SL, Graham C (2013). Sexual compulsivity in heterosexual married adults: the role of sexual excitation and sexual inhibition in individuals not considered “high-risk”. Sex Addict Compuls.

[37] Gullette D, Lyons M (2005). Sexual sensation seeking, compulsivity, and HIV risk behaviors in college students. J Community Health Nurs.

[38] McBride K, Reece M, Sanders S (2008). Using the sexual compulsivity scale to predict outcomes of sexual behavior in young adults. Sex Addict Compuls.

[39] Salmerón-Sánchez P, Ballester-Arnal R, Gil-Llario M, Morell-Mengual V (2015). Sexual compulsivity and sexual sensation seeking: a preliminary approach among male sex workers compared to gay men in Spain. J Sex Marital Therapy.

[40] Chaney M, Dew B (2003). Online experiences of sexually compulsive men who have sex with men. Sex Addict Compuls.

[41] Gil-Llario M, Morell-Mengual V, Giménez-García C, Salmerón-Sánchez P, Ballester-Arnal R (2018). Sexual sensation seeking: a validated scale for spanish gay, lesbian and bisexual people. AIDS Behav.

[42] Muthén LK, Muthén BO (2012). Mplus User’s Guide.

[43] Asparouhov T, Muthén B. Multiple imputation with Mplus. 2010. http://www.statmodel.com/download/Imputations7.pdf.

[44] Whittaker T (2012). Using the modification index and standardized expected parameter change for model modification. J Exp Educ.

[45] Bagozzi R, Yi Y (2011). Specification, evaluation, and interpretation of structural equation models. J Acad Mark Sci.

[46] DiStefano C, Liu J, Jiang N, Shi D (2017). Examination of the weighted root mean square residual: evidence for trustworthiness?. Struct Equ Modeling.

[47] Hooper D, Coughlan J, Mullen M (2008). Structural equation modelling: guidelines for determining model fit. Electron J Bus Res Methods.

[48] Viladrich C, Angulo-Brunet A, Doval E (2017). Un viaje alrededor de alfa y omega para estimar la fiabilidad de consistencia interna. Anal Psicol.

[49] Zhang Z, Yuan K (2015). Robust coefficients alpha and omega and confidence intervals with outlying observations and missing data. Educ Psychol Meas.

[50] Schmitt T, Sass D (2011). Rotation criteria and hypothesis testing for exploratory factor analysis: implications for factor pattern loadings and interfactor correlations. Educ Psychol Meas.

[51] Muthén LK, Muthén BO (2010). Mplus user’s guide.

[52] Worthington R, Whittaker T (2006). Scale development research. Couns Psychol.

[53] Gorsuch R (1983). Factor analysis.

[54] Rosseel Y. lavaan: an R package for structural equation modeling. J Stat Soft. 2012;48(2):1–36. https://www.jstatsoft.org/index.php/jss/article/view/v048i02.

[55] Hu L, Bentler P (1999). Cutoff criteria for fit indexes in covariance structure analysis: conventional criteria versus new alternatives. Struct Equ Model.

[56] Liao W, Lau J, Tsui H, Gu J, Wang Z (2014). Relationship between sexual compulsivity and sexual risk behaviors among chinese sexually active males. Arch Sex Behav.

